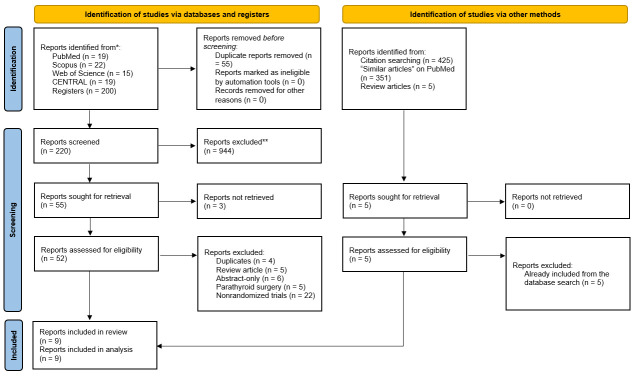# The impact of using near-infrared autofluorescence on parathyroid gland parameters and clinical outcomes during total thyroidectomy: a meta-analytic study of randomized controlled trials

**DOI:** 10.1097/JS9.0000000000001247

**Published:** 2024-03-18

**Authors:** Alaa Safia, Uday Abd Elhadi, Saqr Massoud, Shlomo Merchavy

**Affiliations:** a Department of Otolaryngology, Head and Neck Surgery Unit, Rebecca Ziv Medical Center, Safed, Israel; b True Doctor, Research Wing, Israel

**Keywords:** hypocalcemia, hypoparathyroidism, near-infrared autofluorescence, parathyroid gland, total thyroidectomy

## Abstract

**Background:**

The added benefit of using near-infrared autofluorescence (NIRAF) during total thyroidectomy (TT) remains controversial. This study investigated whether or not NIRAF results in improved patient outcomes postoperatively.

**Materials and methods:**

We analyzed 1711 TT patients, reported in nine randomized controlled trials, following a systematic search of five databases. NIRAF was compared to the standard of care (naked eye with/without white light). Outcomes included parathyroid gland (PG) and calcium parameters and other clinical outcomes. For dichotomous outcomes, the log odds ratio (logOR) was calculated, and for continuous outcomes, the crude mean difference (MD) was measured. Sensitivity analysis was performed when heterogeneity was significant. The revised Cochrane risk of bias tool was used to assess the methodological quality.

**Results:**

Compared to the standard of care, the use of NIRAF was associated with a significant reduction in postoperative hypoparathyroidism [logOR=−0.31; 95% CI: −0.57: −0.05], inadvertent PG removal [logOR=−0.93; 95% CI: −1.60: −0.26], and postoperative hypocalcemia [logOR=−0.43 mmol/l; 95% CI: −0.77: −0.09]. It was also associated with significantly higher postoperative PTH levels [MD=4.78 pg/ml; 95% CI: 2.13: 7.43], PG identification rate [logOR=1.02; 95% CI: 0.31: 1.72], postoperative serum calcium [MD=0.05; 95% CI: 0.00: 0.09], and operative time [MD=9.38 min; 95% CI: 6.68: 12.09]. No difference was seen regarding PG autotransplantation, length of hospital stay, and hospitalization due to hypocalcemia. Seven trials had low risk and the remainder had some concerns.

**Conclusion:**

NIRAF is superior to the naked eye in identifying all four PGs during TT. The reduced risk of postoperative hypoparathyroidism and hypocalcemia reflected this preservation value. However, it was not associated with a change in the length of hospital stay. Although rare, the readmission rate due to hypocalcemia was similar across both methods.

## Introduction

Highlights

- Compared to the standard of care, near-infrared autofluorescence use during total thyroidectomy is associated with a lower risk of postoperative hypoparathyroidism, inadvertent parathyroid gland removal, and hypocalcemia.
- However, it is associated with higher postoperative parathyroid hormone and serum calcium levels, longer operative time, and a greater likelihood of parathyroid gland identification.

Total thyroidectomy (TT) is a common and generally safe procedure; however, it carries inherent risks, notably the potential damage to or inadvertent removal of the parathyroid glands (PGs)^1^. These small, yet critically important glands play a vital role in calcium homeostasis, and their compromise can lead to hypoparathyroidism and subsequent hypocalcemia, conditions characterized by abnormally low levels of parathyroid hormone (PTH) and calcium in the blood, respectively^2^. Hypoparathyroidism and hypocalcemia can manifest with a range of symptoms, from mild paresthesia and muscle cramps to severe cardiac arrhythmias and seizures, necessitating prompt and effective management to mitigate adverse outcomes^3^.

In recent years, near-infrared autofluorescence (NIRAF) has emerged as a novel intraoperative technology aimed at enhancing the visualization of PGs during thyroid surgery^4^. By exploiting the unique autofluorescent properties of parathyroid tissue under near-infrared light, surgeons are afforded real-time, high-contrast imaging, facilitating the identification, and preservation of these glands. This technological advancement holds promise for reducing the incidence of accidental parathyroidectomy or devascularization, and consequently, lowering the risk of postoperative hypoparathyroidism and hypocalcemia^5^.

Despite its potential benefits, the adoption of NIRAF in thyroid surgery is not without challenges and controversies. The variability in equipment, technique, and surgeon experience, coupled with the heterogeneity in patient populations, has resulted in a broad spectrum of reported outcomes. Moreover, the clinical significance of improved PG visualization on long-term patient outcomes, particularly regarding the prevention of hypoparathyroidism and hypocalcemia, remains a subject of ongoing research and debate.

Although several meta-analyses have been conducted so far, all of which included a small number of randomized trials and the main evidence was driven from retrospective cohort studies^5–8^. Additionally, these reviews looked only at the rate of hypocalcemia and hypoparathyroidism without putting into account the PG identification rate, serum calcium level, PTH level, and other clinical outcomes such as operative time, hospitalization rate, and length of hospital stay (LOS).

Therefore, we conducted this investigation of randomized controlled trials (RCTs) to provide conclusive evidence regarding the efficacy of NIRAF in preserving the PGs and in affecting patients’ clinical outcomes following TT.

## Materials and methods

### Design and population

This research has been reported in line with the (Preferred Reporting Items for Systematic Reviews and Meta-Analyses) PRISMA^9^ (Supplemental Digital Content 1, http://links.lww.com/JS9/C130) (Supplemental Digital Content 2, http://links.lww.com/JS9/C131) and (Assessing the Methodological Quality of Systematic Reviews) AMSTAR^10^ (Supplemental Digital Content 3, http://links.lww.com/JS9/C132) Guidelines (Supplemental Material, Supplemental Digital Content 4, http://links.lww.com/JS9/C133). The study protocol was registered on PROSPERO before the conduct of the study. The design of this review followed the PICOS (Population, Intervention, Comparison, Outcome, and Study) framework^11^. We included patients with thyroid pathologies (either benign or malignant) warranting TT. The intervention group included patients who underwent intraoperative PG identification through NIRAF, while the comparison (control) group included patients whose PGs were identified through the standard of care [no NIRAF: naked eye with or without white light (WL)]. The main outcomes included the rates of hypoparathyroidism, hypocalcemia, and PG identification. Only RCTs were considered.

### Literature search

On 24 October 2023, a literature search was performed across four databases (PubMed, Scopus, Web of Science, and Cochrane Registry of Randomized Controlled Trials) and one registry (Google Scholar). In the latter, only the first 200 citations were searched as per the recommendations^12^. A list of relevant keywords and medical terms was used, which were adjusted accordingly as per the searched database. These terms were thyroidectomy AND random* AND near-infrared AND autofluorescence. The full search query, adjusted per searched databases, is provided in (SDC, Table 1, Supplemental Digital Content 4, http://links.lww.com/JS9/C133). Additionally, a manual search was conducted to ensure the quality of the performed database search/screening as well as to identify any other relevant, potentially-missing, studies. This step was done by reviewing the relevant review articles on this topic and by screening the reference list of included studies in our review^13^.

### Selection criteria

Studies meeting the following criteria were included: (a) RCTs; (b) investigated patients who underwent TT; (c) randomized patients into NIRAF and a control group, and (d) reported one of our outcome measures. On the other hand, studies were excluded if: (a) they were single-armed, nonrandomized, or nonexperimental in design; (b) included secondary research (i.e. reviews, opinions, etc.); (c) had duplications; or (d) reported irrelevant outcomes. We did not exclude studies based on the original language of publication.

### Outcomes measures

Outcomes of interest were divided into three main domains: PG parameters, calcium parameters, and clinical parameters. PG parameters included postoperative PTH level, the rate of hypoparathyroidism, PG identification rate, inadvertent PG excision, PG autotransplantation, and hypoparathyroidism warranting treatment. Hypoparathyroidism was defined as a level <20 pg/ml^14^, and it was further subcategorized into permanent (≥6 months) and transient (<6 months) hypoparathyroidism^15,16^. Calcium parameters included postoperative serum calcium level, postoperative hypocalcemia, and postoperative symptomatic hypocalcemia. Hypocalcemia was defined based on a serum level of <8.0 mg/dl^17^. Other clinical parameters included the LOS, operative time (in minutes), and postoperative hospitalization secondary to hypocalcemia.

### Study selection

Studies identified from the literature search were imported into EndNote software, where duplicates were identified and excluded. After that, the remaining studies were exported into an Excel sheet for screening. The screening process was done by two investigators simultaneously over three different phases: title, abstract, and full-text selection. Any differences between them were resolved by consulting the senior author.

### Data curation

The senior author used Microsoft Excel to design the data extraction sheet, which consisted of three worksheet tabs. The first part covered studies’/patients’ characteristics, including studies identification number, authors names, year of publication, country of investigation, study design, sample size, studied population (thyroid pathology; malignant or benign), surgery performed, description of the intervention and control groups, age, sex, and follow-up period. The second part covered our outcomes of interest. The third part covered the methodological quality (risk of bias – ROB) of included RCTs.

### Methodological quality assessment

The revised Cochrane RoB-II tool (revised in 2019) was used to assess the methodological quality of included trials^18^. Each RCTs will be assessed on the level of five domains: randomization, deviation from intended interventions, missing outcome data, outcome measurement bias, and selective reporting. Finally, each trial will be given a quality of low risk of bias, some concerns, or high risk of bias.

### Data analysis

No deviations have been made regarding the predefined analysis plan in our registered protocol. All analyses were conducted per patient except for the PG identification rate, where analyses were done per analyzed gland. The outcome data reported in one study^19^ were in the form of median and interquartile range, and for the purposes of standardizing the reported effect estimate, we converted such data into mean and SD using the equations provided by Luo *et al*.^20^ and Wan *et al*.^21^ Additionally, the serum calcium data were transformed into mmol/l in four RCTs^15,22–24^.

Data analysis was performed using STATA Software (Version 18). For continuous outcomes (i.e. PTH and calcium levels), the crude mean difference (MD) will be calculated along with its 95% CI. For dichotomous outcomes, the log odds ratio (logOR) and its 95% CI will be pooled across studies. The fixed-effects and random-effects models were selected based on the presence of statistical heterogeneity. If heterogeneity was significant (*I*^2^ >50% with *P*-value <0.05), the random-effects model was chosen. Subgroup analyses based on the follow-up period were conducted. If statistical heterogeneity was encountered, a leave-on-out sensitivity analysis was performed to determine if the reported effect estimate was driven by a particular study. The assessment of publication bias was deemed inappropriate due to the lack of enough number of analyzed studies (<10 studies). A *P*-value of <0.05 was deemed statistically significant.

## Results

### Literature search results

**Figure 1:**
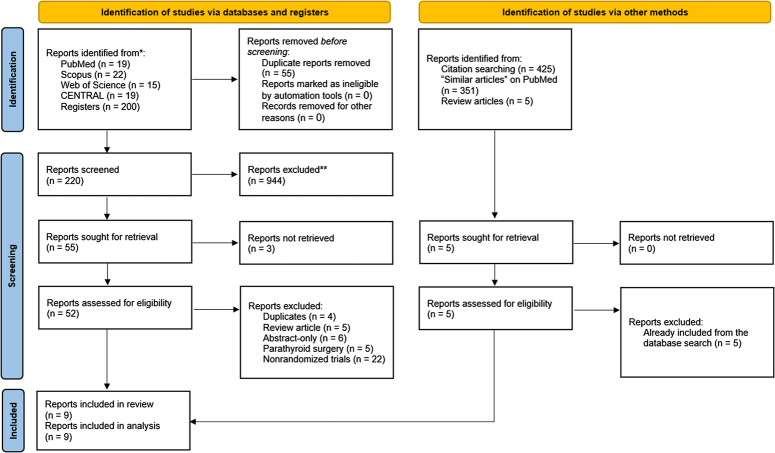
PRISMA diagram showing the results of the literature search and study selection processes.

The results of the literature search and study selection processes are illustrated in Figure 1. In summary, we identified 275 records from the literature search, of which 55 were ruled out as duplicates through EndNote Software. Following the screening of 220 titles and abstracts, only 55 articles were sought for full text retrieval, of which 3 were not accessible. Forty-three articles were then excluded for the following reasons: duplication (four articles), reviews (five articles), abstract-only papers (six articles), parathyroid surgery (five articles), and nonrandomized trials (22 articles). Finally, nine RCTs were deemed eligible for data synthesis^15,16,19,22–27^. No additional results were obtained from the manual search.

### Characteristics of analyzed studies/patients

Nine trials were included, all of which were RCTs. These RCTs were conducted across the globe in Greece (two trials), Argentina (one trial), Denmark (one trial), France (one trial), Germany (one trial), Italy (one trial), China (one trial), and Sweden (one trial). A total of 1711 patients who underwent TT were randomized into the NIRAF (856 patients) and control (855 patients) groups, respectively. As for the indication for TT, all studies included patients with benign and malignant thyroid diseases (eight trials) except for one study that included only patients with benign conditions. The description of the intervention and control groups is provided in Table 1. Regarding sex, males constituted 25.7 and 21.5% of patients in the NIRAF and control groups, respectively. In terms of follow-up, three trials followed patients for 1 day after surgery; three trials followed patients for 6 months; one trial followed patients for 3 months; and one trial followed patients for 12 months.

**Table 1:** Baseline characteristics of studies investigating the use of near-infrared autofluorescence in patients who underwent total thyroidectomy.

			Sample				Method		Age; mean (SD)		Male		FU (month)	
Author (YOP)	Country	Design	NIRAF	No NIRAF	Thyroid pathology	Surgery	NIRAF	No NIRAF	NIRAF	No NIRAF	NIRAF	No NIRAF	NIRAF	No NIRAF
Benmiloud (2020)^17^	France	RCT	121	120	Benign/ Malignant	TT	NIRAF (Fluobeam 800)	WL + NE	52.5 (46–63)	51 (45–64)	25	24	6	6
Yin (2022)^14^	China	RCT	90	90	Benign/ Malignant	TT	NIRAF (handheld laser probe, a camera, and a visualization system with two light sources)	WL + NE	42.1 (9.5)	42.7 (9.1)	20	16	6	6
Lykke (2023)^24^	Denmark	RCT	69	78	Benign/ Malignant	Primary TT/CTT	NIRAF (Elevision IR + Fluobeam 800) + WL	WL + NE	57.4 (16)	52.2 (15.8)	19	18	3	3
Bergenfelz (2023)^23^	Sweden	RCT	246	240	Benign/ Malignant	TT	NIRAF (Fluobeam LX)	WL + NE	50.2 (14)	51.4 (15.9)	46	51	1 day	1 day
Wolf (2022)^25^	Germany	RCT	30	30	Benign	TT	NIRAF (Tricam SL)	WL + NE	57 (12.7)	59 (11.9)	9	8	1 day	1 day
Dip (2019)^20^	Argentina	RCT	85	85	Benign/ Malignant	Primary TT	NIRAF (Fluobeam 800) + WL	WL + NE	47.3 (13.6)	45.8 (13.7)	44	26	6	6
Papavramidis (2021)^21^	Greece	RCT	90	90	Benign/ Malignant	TT	NIRAF (Fluobeam LX)	WL + NE	48.4 (14.6)	45.73 (13.6)	27	20	1 day	1 day
Tzikos (2022)^13^	Greece	RCT	25	22	Benign/ Malignant	TT	NIRAF (Fluobeam LX)	WL + NE	49.4 (14.6)	46.73 (13.6)	8	5	12	12
Rossi (2023)^22^	Italy	RCT	100	100	Benign/ Malignant	TT	NIRAF (Fluobeam LX)	WL + NE	44.52 (11.22)	44.82 (10.13)	22	16	6	6

CTT, complete total thyroidectomy; FU, follow-up; NE, naked eye; NIRAF, near-infrared autofluorescence; RCT, randomized controlled trial; SD, standard deviation; TT, total thyroidectomy; WL, white light; YOP, year of publication.

### Methodological quality results

The summary of the methodological quality of each analyzed RCT in each of the assessed domains is provided in Table 2. Overall, seven RCTs^15,16,19,23,25–27^ had low risk of bias while the remaining two trials had some concerns^22,24^, mostly associated with the selective reporting of outcomes (mainly due to the lack of a registered protocol).

**Table 2:** Risk of bias assessment of included randomized controlled trials using the revised Cochrane risk of bias tool.

Author (YOP)	Randomization process	Deviations from intended interventions	Missing outcome data	Measurement of outcome	Selection of reported results	Overall bias
Benmiloud (2020)^17^	Low	Low	Low	Low	Low	Low
Yin (2022)^14,25^	Low	Low	Low	Low	Low	Low
Lykke (2023)^24^	Low	Low	Low	Low	Low	Low
Bergenfelz (2023)^23^	Low	Low	Low	Low	Low	Low
Wolf (2022)^25^	Low	Low	Low	Low	Low	Low
Dip (2019)^20^	Low	Low	Low	Low	Some concerns	Some concerns
Papavramidis (2021)^21^	Low	Low	Low	Low	Low	Low
Tzikos (2022)^13^	Low	Low	Low	Low	Low	Low
Rossi (2023)^22^	Low	Low	Low	Low	Some concerns	Some concerns

YOP, year of publication.

### PG parameters

#### PTH level (pg/ml)

**Figure 2:**
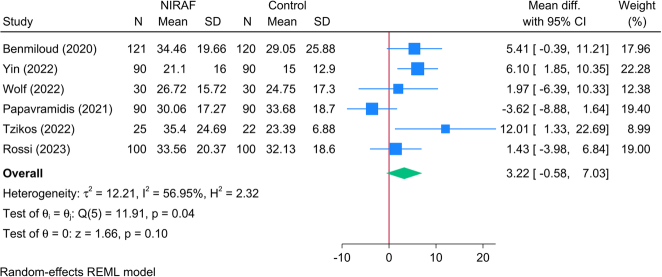
Forest plot showing the difference in postoperative parathyroid hormone levels between near-infrared autofluorescence and standard of care.

**Figure 3:**
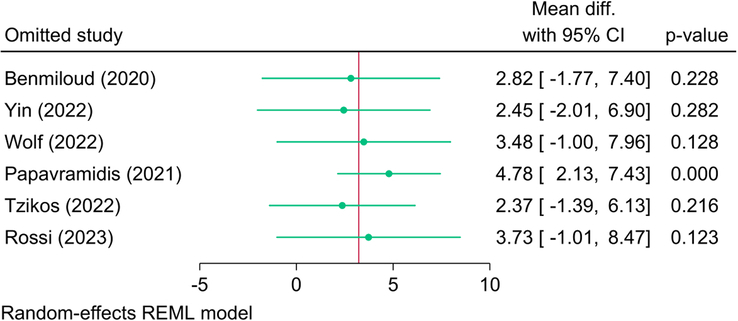
Leave-one-out sensitivity analysis of postoperative parathyroid hormone level between near-infrared autofluorescence and standard of care.

Six RCTs (908 patients) investigated the serum level of PTH, and the pooled meta-analysis revealed no significant change in the reported PTH level between NIRAF and the control group [MD=3.22; 95% CI: −0.58: 7.03] (Fig. 2). The analysis revealed significant statistical heterogeneity [*I*^2^=56.95%, *P*=0.04]; however, the leave-one-out sensitivity analysis revealed a significant difference between both groups (in favor of the NIRAF group) following the exclusion of the study of Papavramidis *et al*.^23^ [MD=4.78; 95% CI: 2.13: 7.43] (Fig. 3). The subgroup analysis of PTH level based on follow-up showed no significant effect modification of time on PTH parameter (*P*=0.19) with six studies investigating PTH levels on postoperative day 1 with just one study following patients for 6 or 12 months, respectively.

#### Postoperative hypoparathyroidism rate

**Figure 4:**
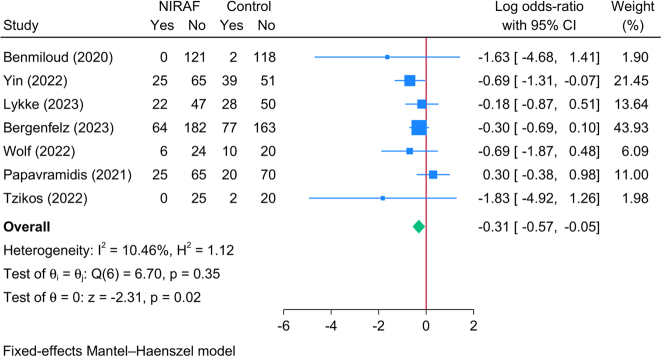
Forest plot showing the difference in postoperative hypoparathyroidism between near-infrared autofluorescence and standard of care.

Seven RCTs (1341 patients) reported the rate of postoperative hypoparathyroidism, and the pooled meta-analysis revealed a significantly lower risk of hypoparathyroidism in patients who underwent NIRAF as compared to the control group [logOR=−0.31; 95% CI: −0.57: −0.05] (Fig. 4). There was no significant statistical heterogeneity encountered [*I*^2^=10.46%, *P*=0.35]. Compared to the control group, patients who underwent NIRAF had significantly lower risk of transient hypoparathyroidism [logOR=−0.28; 95% CI: −0.54: −0.01]; however, no significant difference was observed for permanent hypoparathyroidism (SDC, Fig. 1, Supplemental Digital Content 4, http://links.lww.com/JS9/C133).

#### Hypoparathyroidism rate warranting treatment

Three RCTs (987 patients) investigated the risk of hypoparathyroidism warranting treatment in patients undergoing TT. The meta-analysis revealed no significant change in the risk of treatment need between both NIRAF and control groups, regarding both calcium or calcium plus vitamin D administration (SDC, Fig. 2, Supplemental Digital Content 4, http://links.lww.com/JS9/C133).

#### PG identification rate

**Figure 5:**
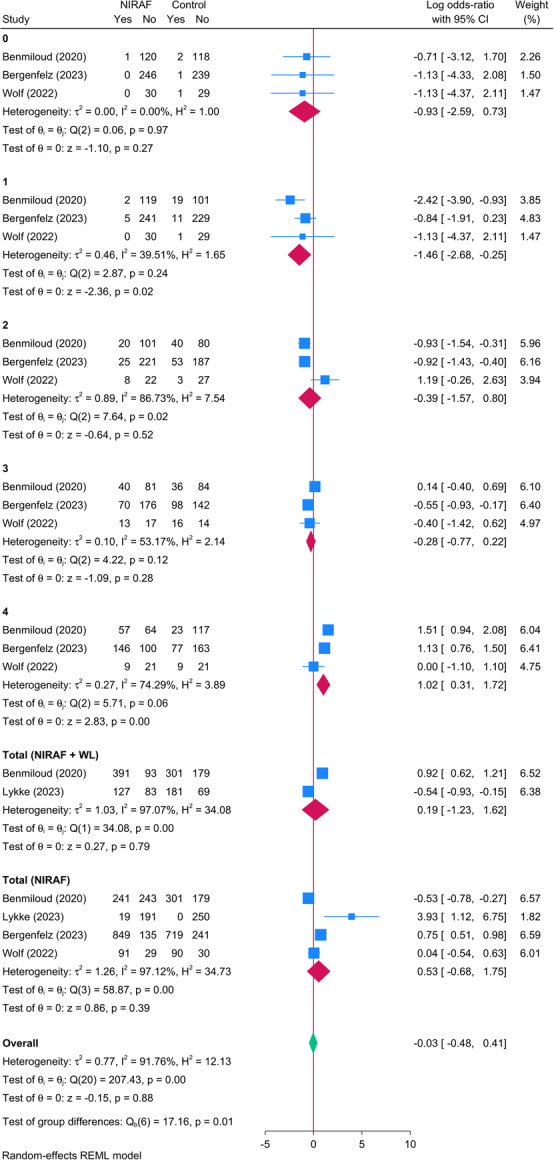
Forest plot showing the difference in parathyroid gland identification rate between near-infrared autofluorescence and standard of care.

Four RCTs (3288 PGs) reported the rate of PG identification among patients undergoing TT. Compared to the control group, the use of NIRAF alone or combined with WL did not result in any significant change in the identification rate of PG. However, in patients with one identified PG, the use of NIRAF resulted in significantly lower odds than control in identifying these glands [three studies, logOR=−1.46; 95% CI: −2.68: −0.25]. Controversially, in patients with four PGs, the use of NIRAF was associated with greater likelihood of identifying these PGs [three studies, logOR=1.02; 95% CI: 0.31: 1.72] (Fig. 5).

#### Inadvertent PG excision

**Figure 6:**
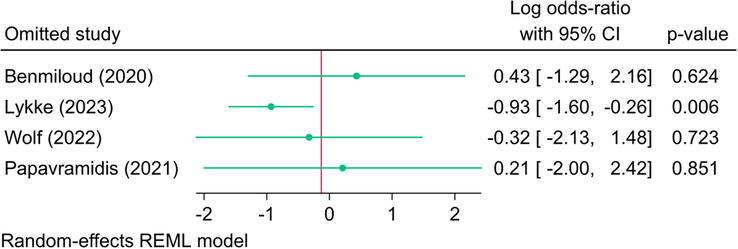
Leave-one-out sensitivity analysis of inadvertent parathyroid gland removal between near-infrared autofluorescence and standard of care.

Four RCTs (627 patients) reported the rate of unintentional/inadvertent excision or removal of PGs during TT, and the meta-analysis revealed no significant difference in the risk of inadvertent PG excision between NIRAF and control groups [logOR=-0.13; 95% CI: −1.68: 1.42] (SDC, Fig. 3, Supplemental Digital Content 4, http://links.lww.com/JS9/C133). There was significant statistical heterogeneity [*I*^2^=81.03%, *P*=0.001. However, the meta-analysis revealed a significant reduction in the risk of inadvertent removal of PGs with the use of NIRAF as compared to controls following the exclusion of the study of Lykke *et al*.^26^ [logOR=−0.93; 95% CI: −1.60: −0.26] (Fig. 6).

#### PG autotransplantation

Three RCTs (927 patients) reported the rate of PG autotransplantation in patients undergoing TT. The meta-analysis revealed no significant change in the risk of PG autotransplantation between the NIRAF and control groups [logOR=−0.11; 95% CI: −1.38: 1.17] (SDC, Fig. 4, Supplemental Digital Content 4, http://links.lww.com/JS9/C133). Although significant statistical heterogeneity was observed [*I*^2^=83.40%, *P*=0.01], the sensitivity analysis revealed no significant change in the reported effect estimate.

### Calcium parameters

#### Postoperative serum calcium level (mmol/l)

**Figure 7:**
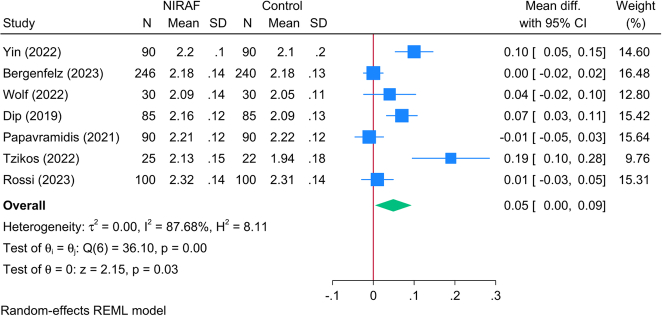
Forest plot showing the difference in postoperative serum calcium levels between near-infrared autofluorescence and standard of care.

Seven RCTs (1323 patients) reported the postoperative serum levels of calcium following TT. The meta-analysis revealed that patients who underwent NIRAF had significantly higher serum calcium levels compared to controls [MD=0.05; 95% CI: 0.00: 0.09] (Fig. 7). A significant statistical heterogeneity was noted [*I*^2^=87.68%; *P*=0.001]; however, the leave-one-out sensitivity analysis showed significant changes in the reported effect estimate with the removal of one trial at a time (SDC, Fig. 5, Supplemental Digital Content 4, http://links.lww.com/JS9/C133). The data on the effect of NIRAF over different follow-up period was inconclusive since most studies investigated the serum calcium of these patients on postoperative day one with only one study assessing serum calcium level at 6 and 12 months (SDC, Fig. 6, Supplemental Digital Content 4, http://links.lww.com/JS9/C133).

#### The rate of postoperative hypocalcemia

**Figure 8:**
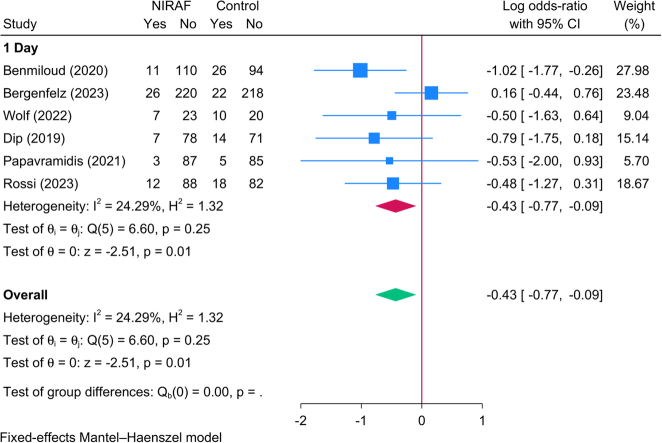
Forest plot showing the difference in postoperative hypocalcemia between near-infrared autofluorescence and standard of care.

Six RCTs (1337 patients) reported the rate of postoperative hypocalcemia following TT. The meta-analysis revealed a significant reduction in the risk of hypocalcemia in patients who underwent NIRAF compared to the control group [logOR=−0.43; 95% CI: −0.77: −0.09] (Fig. 8). This finding was conclusive given the lack of significant statistical heterogeneity [*I*^2^=24.29%, *P*=0.25]. Also, it should be noted that these data are reflective of findings on postoperative day 1 with no other follow-up assessments.

#### The rate of postoperative symptomatic hypocalcemia

**Figure 9:**
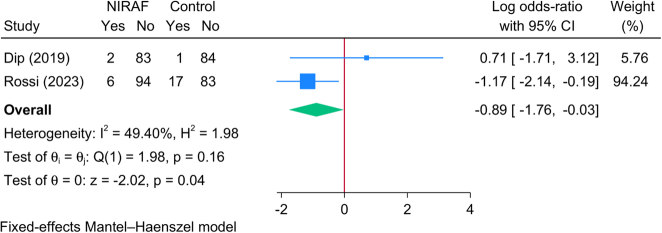
Forest plot showing the difference in postoperative symptomatic hypocalcemia between near-infrared autofluorescence and standard of care.

Two RCTs (370 patients) reported the rate of symptomatic postoperative hypocalcemia among patients who underwent TT. The meta-analysis revealed that the risk of postoperative symptomatic hypocalcemia was significantly lower in patients who underwent NIRAF compared to the control group [logOR=−0.89; 95% CI: −1.76: −0.03] (Fig. 9). No significant statistical heterogeneity was encountered [*I*^2^=49.40%, *P*=0.16].

### Other Clinical Parameters

#### LOS (days)

Four RCTs (987 patients) reported the LOS in patients undergoing TT. The meta-analysis revealed no significant change in the LOS between patients who underwent NIRAF or those in the control group [MD=0.05 days; 95% CI: −0.06: 0.16]. No statistical heterogeneity was observed [*I*^2^=0.0%, *P*=0.90] (SDC, Fig. 7, Supplemental Digital Content 4, http://links.lww.com/JS9/C133).

#### Operative time (minutes)

**Figure 10:**
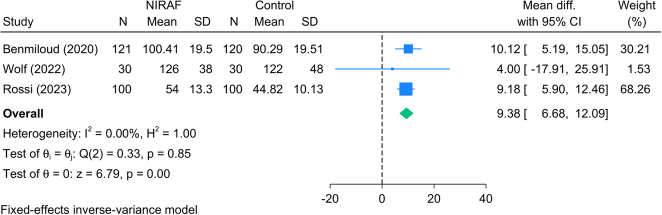
Forest plot showing the difference in operative time between near-infrared autofluorescence and standard of care.

Three RCTs (501 patients) reported the operative time in patients undergoing TT. The meta-analysis revealed that patients who underwent NIRAF had significantly higher operative time compared to the control group [MD=9.38 min; 95% CI: 6.68: 12.09]. No statistical heterogeneity was observed [*I*^2^=0.0%, *P*=0.85] (Fig. 10).

#### The rate of postoperative hospitalization secondary to hypocalcemia

Two RCTs (656 patients) reported the rate of postoperative hospitalization due to hypocalcemia in patients undergoing TT. The meta-analysis revealed no significant change in the risk of postoperative hospitalization between patients who underwent NIRAF or those in the control group [logOR=0.68; 95% CI: −0.16: 1.52] (SDC, Fig. 8, Supplemental Digital Content 4, http://links.lww.com/JS9/C133). No statistical heterogeneity was observed [*I*^2^=0.0%, *P*=0.76].

## Discussion

This meta-analysis, comprising nine RCTs with a total of 1711 patients, represents a comprehensive evaluation of the efficacy of NIRAF in enhancing the outcomes of TT by providing better visualization of the PGs compared to the standard of care (visualization with the naked eye with/out WL). The results unequivocally demonstrate that NIRAF significantly improves several key parameters associated with PG preservation and function.

### PTH levels and hypoparathyroidism rates

Our findings regarding PTH levels suggest a nuanced role for NIRAF in preserving parathyroid function. The lack of a significant change in overall PTH levels between the NIRAF and control groups might initially imply that NIRAF does not dramatically impact parathyroid function. However, the sensitivity analysis revealing a difference upon the exclusion of one study suggests a potential for study-specific factors or patient characteristics to influence these outcomes. This could indicate that NIRAF’s effectiveness might vary based on surgical technique, patient anatomy, or the inherent sensitivity of the PGs in different individuals. It is crucial for future research to explore these variables to determine under what circumstances NIRAF most effectively preserves parathyroid function.

The significant reduction in the rate of postoperative hypoparathyroidism in the NIRAF group is a pivotal finding. It implies that NIRAF aids in better visualization and, consequently, the preservation of the PG during surgery. This is particularly relevant for transient hypoparathyroidism, which can be a common complication after thyroidectomy. Our findings are in line with a previous meta-analytical study conducted in 2021 which included eight studies (three randomized and five observational studies). The authors found that the rate of transient postoperative hypoparathyroidism was significantly lower in the NIRAF group (28.31 vs. 33.36%); however, no data regarding the comparative risk was provided which could have been provided more clinically-meaningful results^6^. The ability of NIRAF to reduce this complication has substantial implications for patient care, potentially leading to fewer postoperative complications, reduced need for treatment of hypoparathyroidism, and overall better quality of life for patients’ postsurgery.

### PG identification and excision

The differential impact of NIRAF on the identification of PGs is particularly intriguing. Based on our findings, the improved odds of identifying all four glands suggest that NIRAF might enhance the surgeon’s ability to detect these glands, especially when they are anatomically well-differentiated. This goes in line with our finding that the naked eye (control group) is more likely to identify only one PG; a drawback that can be extra challenging in unusual surgical scenarios or in instances where the glands are not distinctly separate. This finding might also hint at the potential for NIRAF to help distinguish between PGs and other tissues in complex cases, thereby improving surgical precision. These findings are novel and have not been discussed in any of the previously published meta-analyses on this topic^5–8^, except for the inadvertent parathyroidectomy (unintentional removal of the PGs), where previous reviews have shown either significantly reduced risk^5,7^ or prevalenc^6^ associated with NIRAF use.

The finding regarding inadvertent PG excision highlights the potential for NIRAF to reduce surgical errors. The initial lack of significant difference, followed by a notable reduction in risk upon the exclusion of a specific study, suggests that NIRAF’s effectiveness in preventing inadvertent excision may depend on specific surgical settings or techniques. This could be influenced by factors such as the surgeon’s experience, the complexity of the surgical field, or the anatomical variability among patients. Understanding these factors could help optimize the use of NIRAF in surgeries where the risk of inadvertent excision is higher.

### Calcium parameters

Our analysis indicated improvement in postoperative serum calcium levels in the NIRAF group which is a key outcome, suggesting a protective effect against the development of hypocalcemia, a common and concerning complication after thyroidectomy. This is in line with previous meta-analyses which have shown reduced risk^5,8^ and prevalence^6^ of hypocalcemia, of the transient type, following the use of NIRAF compared to the standard of care (relative risk=0.49; 95% CI: 0.35: 0.68). This finding indicates that NIRAF not only assists in the visual identification of PGs but also contributes to their functional preservation. The reduction in both the overall and symptomatic rates of postoperative hypocalcemia further supports the clinical utility of NIRAF. By reducing the incidence of hypocalcemia, NIRAF could potentially decrease the need for postoperative calcium supplementation, reduce patient discomfort, and lower the risk of severe complications associated with hypocalcemia.

### Other clinical parameters

In our study, we noted that the LOS did not differ based on the intervention used to visualize the PGs. While NIRAF does not significantly impact the LOS, this might be attributed to various postoperative care protocols that are not directly influenced by NIRAF use. It is important to consider that the length of stay could be affected by a range of factors, including hospital policies, patient comorbidities, and other aspects of postoperative care. As these factors play important roles into patient care and determining patient outcomes, these findings are novel and have not been discussed in previous research.

The increased operative time observed with NIRAF use raises important considerations. While longer surgeries may be seen as a disadvantage, this increase in time might be a trade-off for more careful and meticulous surgery, leading to better outcomes in terms of PG preservation. Furthermore, the observed difference in operative time between NIRAF and the standard of care although statistically significant, its clinical applicability is questioned since this difference accounts for only 9 min between both visualization methods, which could be further attributed to surgical protocol rather than the visualization procedure itself. The decision to use NIRAF should thus consider not only the direct surgical outcomes but also the broader implications for patient recovery and long-term health.

### Study limitations and future research

Several limitations were encountered, the most important of which is the potential effect-modifying role of the surgeon’s experience. Although most studies (seven out of nine RCTs) indicated that all procedures were performed by experienced surgeons, the level of expertise varied substantially. For instance, in one trial, experienced surgeons were defined as those performing >25 procedures/year^19^; however, that number was higher in other studies reaching >100 procedures/year^24^ or >200 procedures/surgeon^27^. Additionally, some studies reported that a prior NIRAF camera training was implemented to avoid a learning curve^22,26^, while others did not. Another limitation is the overreliance on postoperative day (POD) one as a reliable indicator of absolute hypocalcemia postoperatively, when it is known that calcium levels can change beyond that time limit^28^. Additionally, the significant heterogeneity in some analyses (e.g. PTH levels and inadvertent PG excision) underscores the importance of considering individual study designs and patient populations in interpreting these results. For example, one of the analyzed RCTs included patients with benign thyroid conditions, while the remaining trials included patients with combined malignant/benign conditions. Whether or not the indication of surgery could play an effect-modifying role on patients’ outcomes in yet to be investigated. Future research should aim to standardize methodologies and patient selection criteria to provide more uniform data. The variation in follow-up periods across studies, particularly for serum calcium levels, suggests the need for longer-term studies to fully understand NIRAF’s impact on sustained parathyroid function.

## Conclusion

Our findings support the superiority of NIRAF to the naked eye in identifying all four PGs during TT. The reduced risk of postoperative hypoparathyroidism and hypocalcemia reflected this preservation value. However, it was not associated with a change in the LOS. Although rare, the readmission rate due to hypocalcemia was similar across both methods.

## Ethical approval

Not applicable. This is a systematic review paper.

## Consent

Not applicable. This is a systematic review paper.

## Sources of funding

This research received no funding from any institutional or non-institutional organizations.

## Author contribution

A.S.: conceptualization, project administration, and investigation; S.M. and U.A.E.: data curation and methodology; S.M.: formal analysis; Funding acquisition: not applicable; S.M.: resources, software, and supervision; S.M. and A.S.: validation; U.A.E.: writing – original draft; A.S. and S.M.: writing – review and editing; A.S.: data analysis and interpretation.

## Conflicts of interest disclosure

The authors declare no competing interests associated with the conduct of this work.

## Research registration unique identifying number (UIN)

CRD42023476967.

## Guarantor

Alaa Safia.

## Data availability statement

The data used and analyzed in this study can be made available upon reasonable request by contacting the corresponding author.

## Provenance and peer review

Not commissioned, externally peer-reviewed.

## Supplementary Material

**Figure:**