# Supplementary material for: The impact of using near-infrared autofluorescence on parathyroid gland parameters and clinical outcomes during total thyroidectomy: a meta-analytic study of randomized controlled trials
Source: Int J Surg. 2024 Mar 18;110(6):3827–38. doi: 10.1097/JS9.0000000000001247 (PMC11175777; doi:10.1097/JS9.0000000000001247)
Supplement: Supplementary file 3 [file js9-110-3827-s004.docx]

**The Impact of Using Near-Infrared Autofluorescence on Parathyroid Gland Parameters and Clinical Outcomes During Total Thyroidectomy: A Meta-Analytic Study of Randomized Controlled Trials**

**SDC, Table 1**. The search query employed in each database searched in our review

| Database | No | Search Query | Results |
| --- | --- | --- | --- |
| PubMed | | | |
|  | #1 | Thyroidectomy[tiab] OR thyroidectomies[tiab] OR "Thyroidectomy"[Mesh] OR “thyroid surgery”[tiab] OR “thyroid and parathyroid surgery”[tiab] OR “thyroid lobectomy”[tiab] | 36365 |
|  | #2 | Random*[tiab] OR "Randomized Controlled Trial" [Publication Type] | 1592349 |
|  | #3 | “Near-infrared”[tiab] OR NIR[tiab] | 66202 |
|  | #4 | Autofluorescence[tiab] OR fluorescence[tiab] OR "Fluorescence"[Mesh] | 414883 |
|  | #5 | #3 AND #4 | 12267 |
|  | #6 | NIR-AF[tiab] OR NIRAF[tiab] OR NIRL[tiab] | 148 |
|  | #7 | #5 OR #6 | 12285 |
|  | #8 | #1 AND #2 AND #7 | 19 |
| Scopus | | | |
|  | #1 | TITLE-ABS-KEY(Thyroidectomy) OR TITLE-ABS-KEY(thyroidectomies) OR TITLE-ABS-KEY(“thyroid surgery”) OR TITLE-ABS-KEY(“thyroid and parathyroid surgery”) OR TITLE-ABS-KEY(“thyroid lobectomy”) | 53063 |
|  | #2 | TITLE-ABS-KEY(Random*) | 3185717 |
|  | #3 | TITLE-ABS-KEY(“Near-infrared”) OR TITLE-ABS-KEY(NIR) | 206441 |
|  | #4 | TITLE-ABS-KEY(Autofluorescence) OR TITLE-ABS-KEY(fluorescence) | 1060831 |
|  | #5 | #3 AND #4 | 24604 |
|  | #6 | TITLE-ABS-KEY(NIR-AF) OR TITLE-ABS-KEY(NIRAF) OR TITLE-ABS-KEY(NIRL) | 171 |
|  | #7 | #5 OR #6 | 24638 |
|  | #8 | #1 AND #2 AND #7 | 22 |
| Web of Science | | | |
|  | #1 | AB=Thyroidectomy OR AB=thyroidectomies OR AB=“thyroid surgery” OR AB=“thyroid and parathyroid surgery” OR AB=”thyroid lobectomy” | 18379 |
|  | #2 | AB=Random* | 2037881 |
|  | #3 | AB=“Near-infrared” OR AB=NIR | 156871 |
|  | #4 | AB=Autofluorescence OR AB=fluorescence | 539127 |
|  | #5 | #3 AND #4 | 15996 |
|  | #6 | AB=NIR-AF OR AB=NIRAF OR AB=NIRL | 145 |
|  | #7 | #5 OR #6 | 16021 |
|  | #8 | #1 AND #2 AND #7 | 15 |
| CENTRAL | | | |
|  | #1 | Thyroidectomy OR thyroidectomies OR “thyroid surgery” OR “thyroid and parathyroid surgery” OR “thyroid lobectomy” | 2484 |
|  | #2 | Thyroidectomy[MeSH] | 822 |
|  | #3 | #1 OR #2 | 2484 |
|  | #4 | Random* | 1345331 |
|  | #5 | “Near-infrared” OR NIR | 428 |
|  | #6 | Autofluorescence OR fluorescence | 4963 |
|  | #7 | #5 AND #6 | 73 |
|  | #8 | NIR-AF OR NIRAF OR NIRL | 19 |
|  | #9 | #7 OR #8 | 89 |
|  | #10 | #3 AND #4 AND #9 | 19 |
| Google Scholar | | | |
|  | With all of the words | Thyroid random near-infrared autofluorescence | - |
|  | With the exact phrase | - | - |
|  | With at least one of the words | - | - |
|  | Total | As per recent guidelines, the first 200 records were selected | 200 |

**
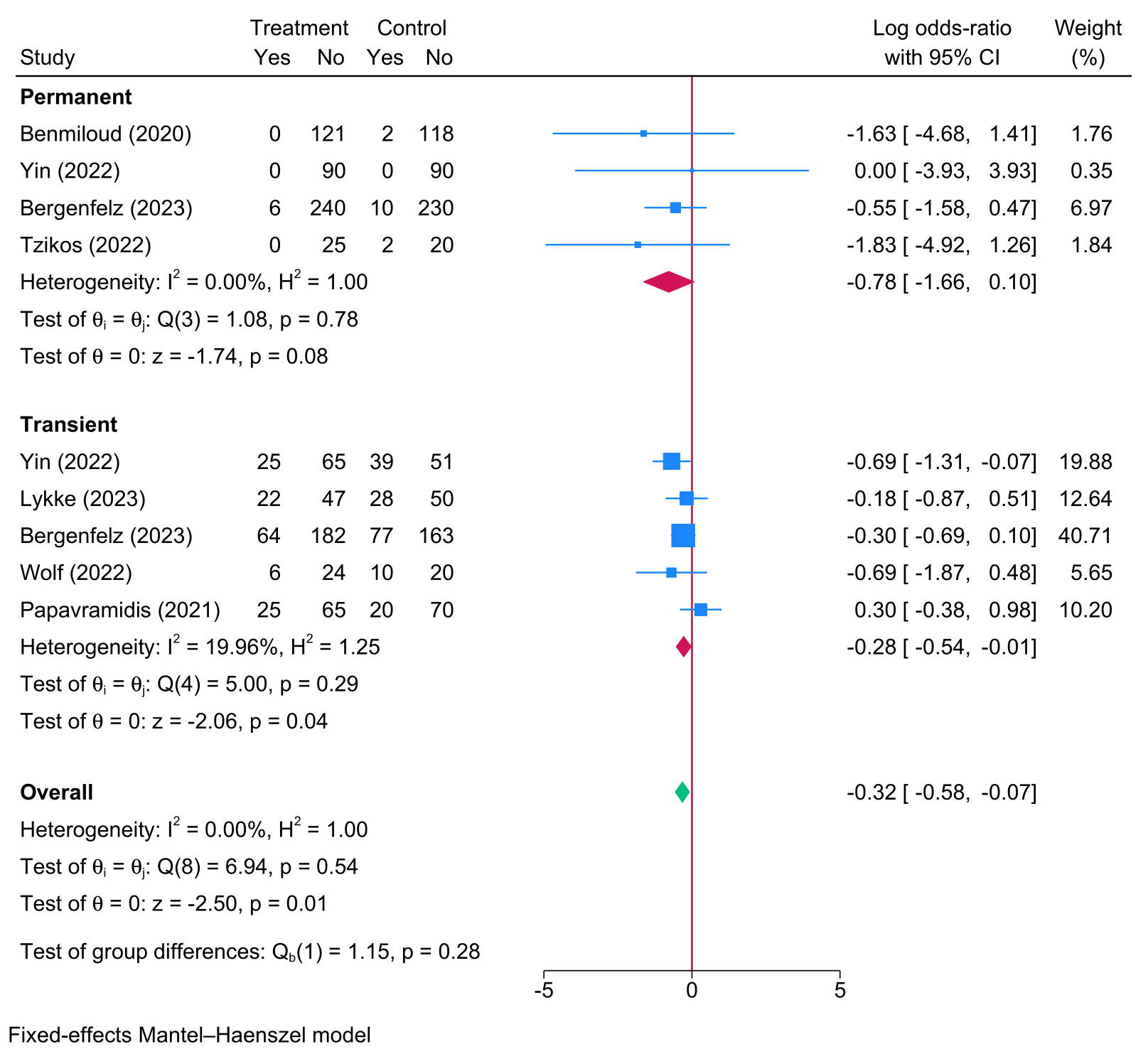
**

**SDC, Figure 1.** Forest plot showing the difference in postoperative transient/permanent hypoparathyroidism between near-infrared autofluorescence and standard of care.

**
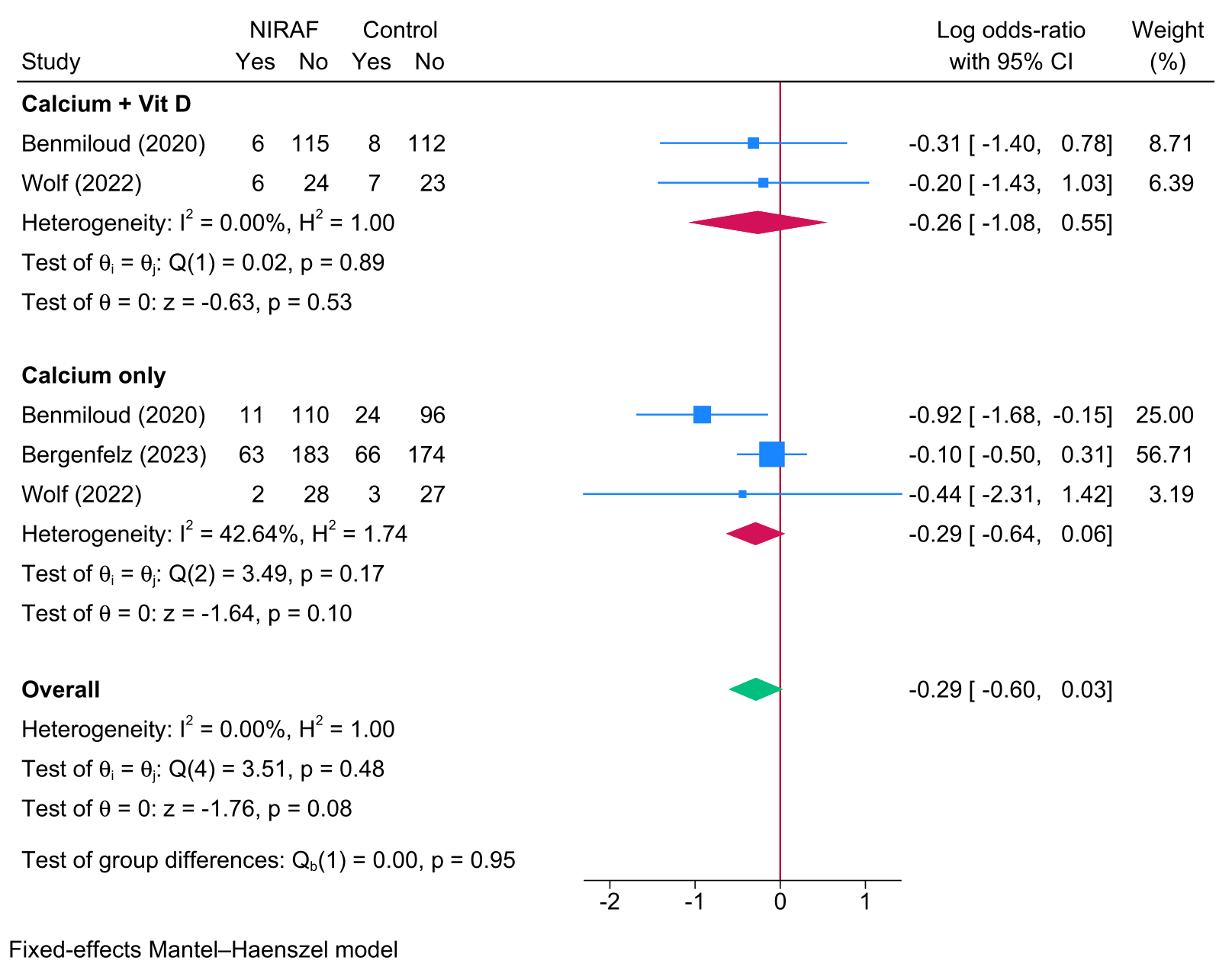
**

**SDC, Figure 2.** Forest plot showing the difference in postoperative hypoparathyroidism warranting treatment between near-infrared autofluorescence and standard of care.

**
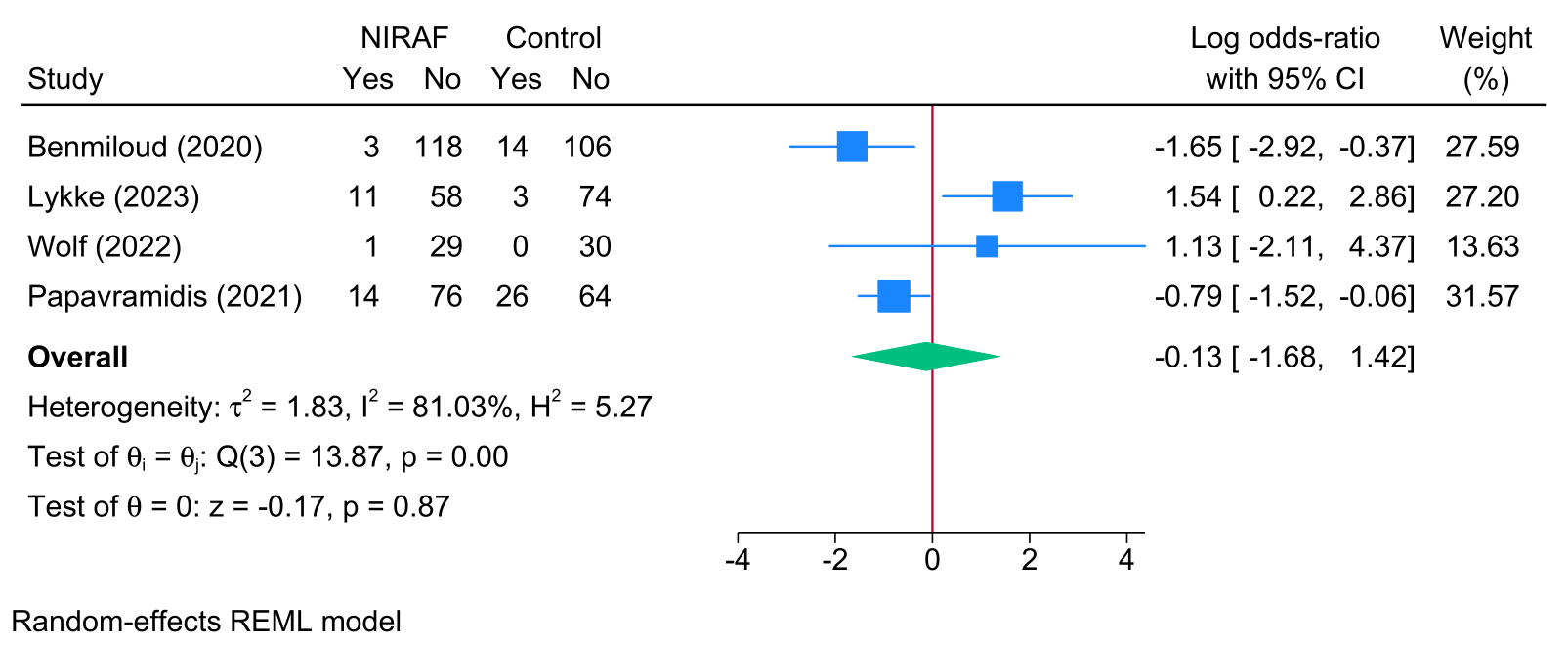
**

**SDC, Figure 3.** Forest plot showing the difference in inadvertent parathyroid gland removal between near-infrared autofluorescence and standard of care.

**
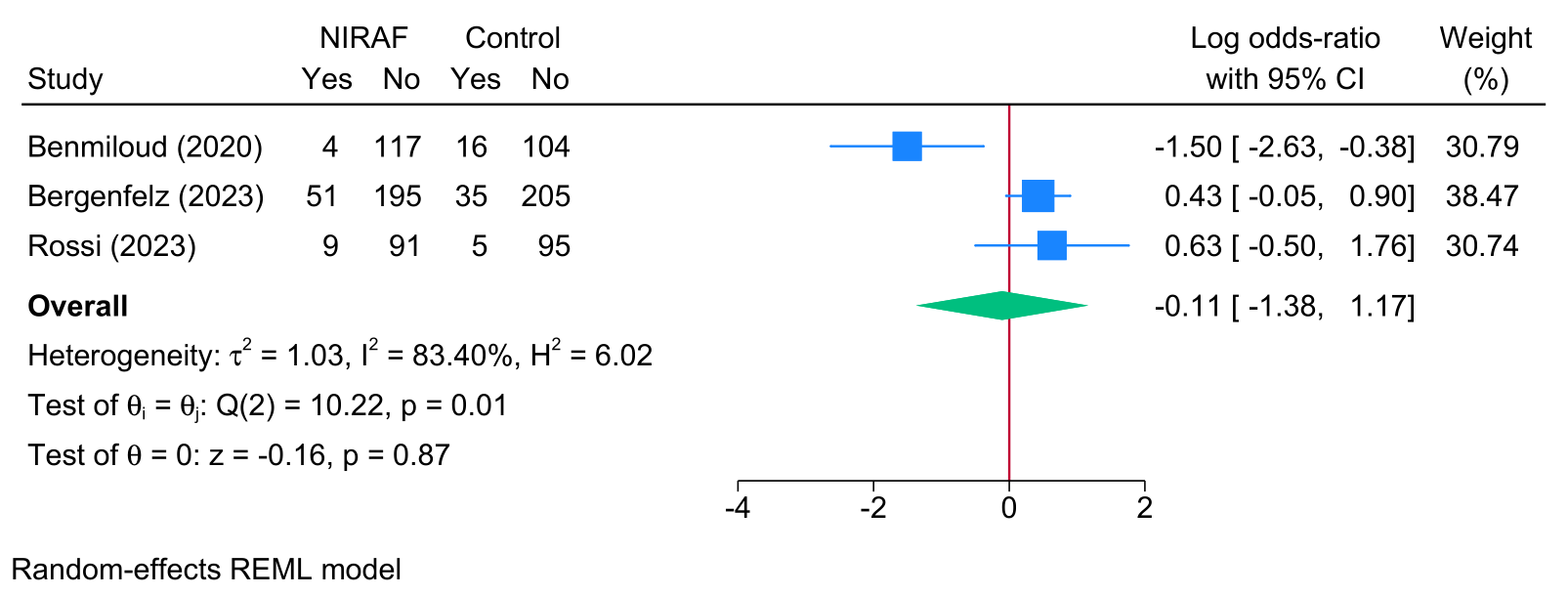
**

**SDC, Figure 4.** Forest plot showing the difference in parathyroid gland autotransplantation between near-infrared autofluorescence and standard of care.

**
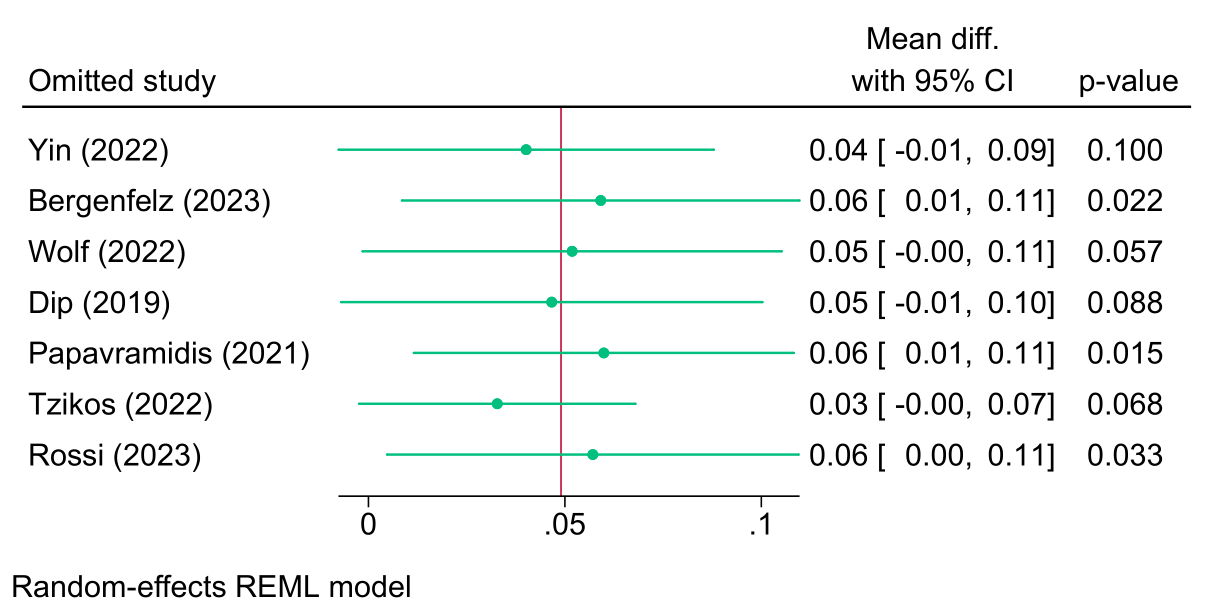
**

**SDC, Figure 5.** Leave-one-out sensitivity analysis of postoperative serum calcium level between near-infrared autofluorescence and standard of care.

**
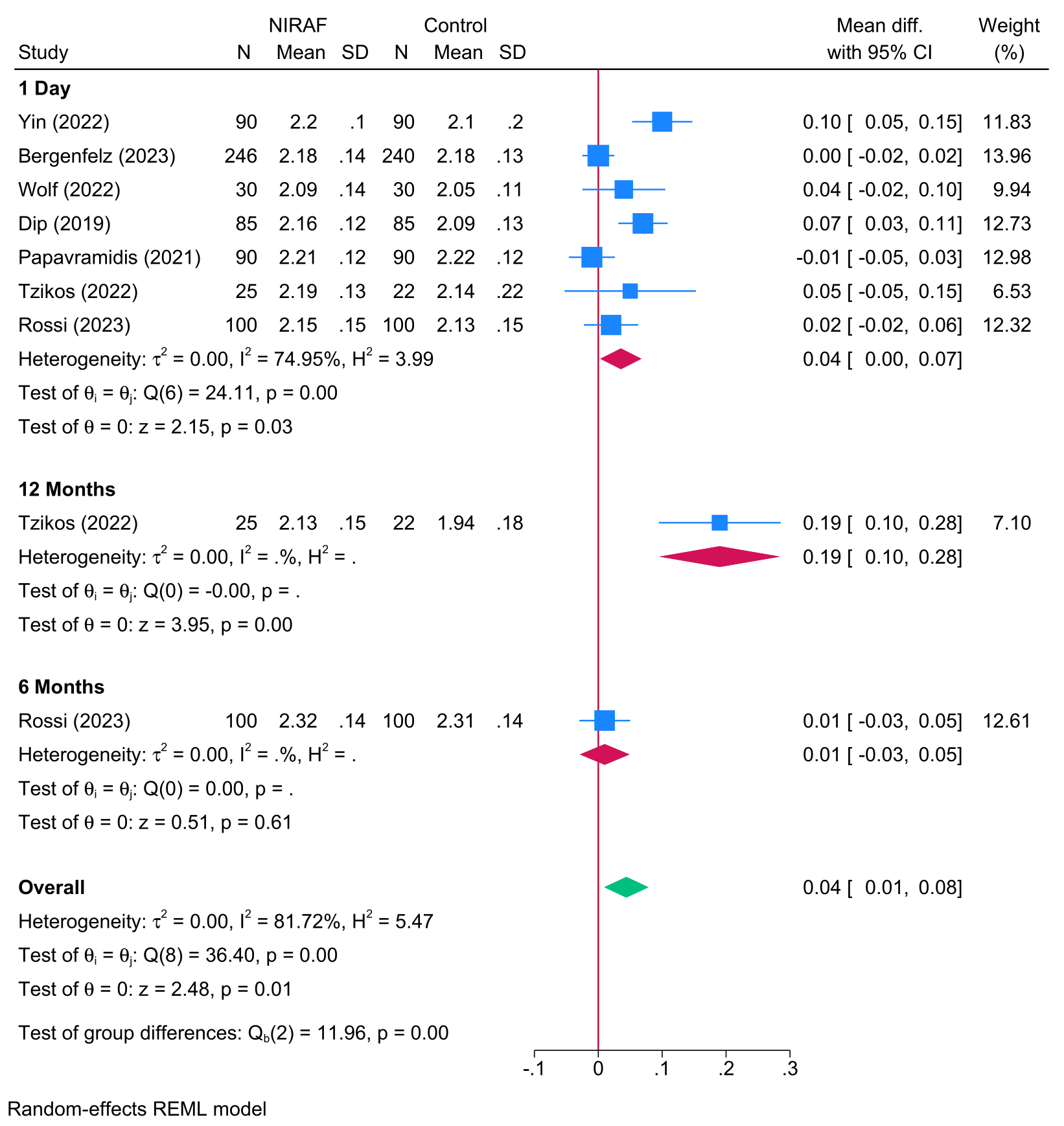
**

**SDC, Figure 6.** Forest plot showing the difference in postoperative serum calcium levels between near-infrared autofluorescence and standard of care, stratified by the follow-up time.

**
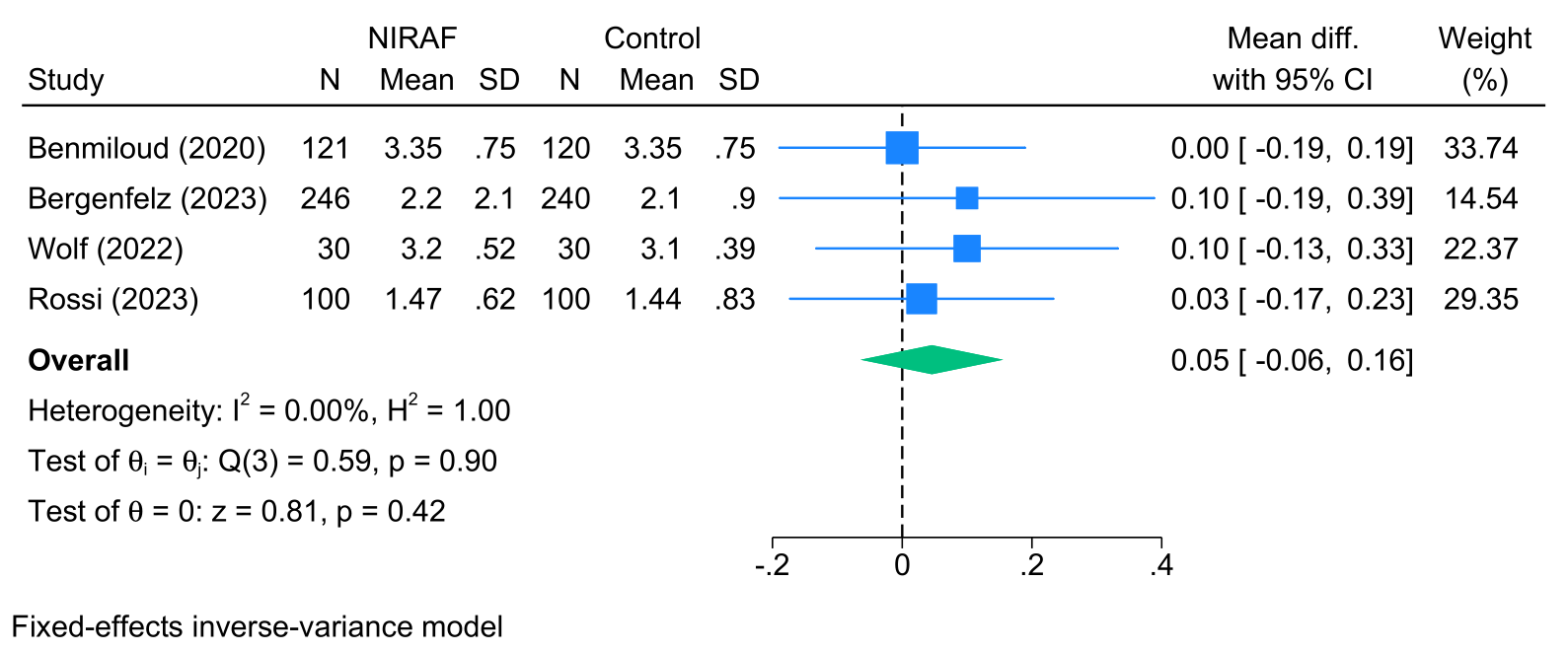
**

**SDC, Figure 7.** Forest plot showing the difference in length of hospital stay between near-infrared autofluorescence and standard of care.

**
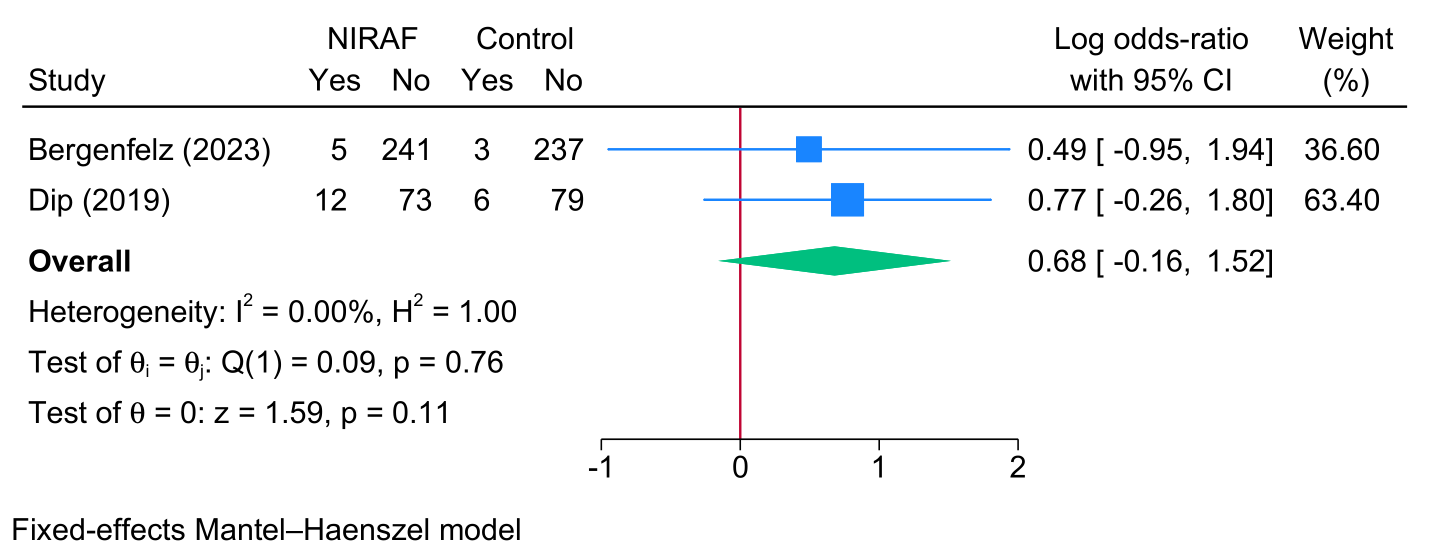
**

**SDC, Figure 8.** Forest plot showing the difference in hospitalization rate due to hypocalcemia between near-infrared autofluorescence and standard of care.
